# Crown Work

**Published:** 1885-01

**Authors:** A. M. Ross

**Affiliations:** Chicopee, Mass.


### CROWN WORK.

BY DR. A. M. ROSS, CHICOPEE, MASS.

In dental mechanics there is no subject that has received greater attention during the past few years than that of tooth crowning. It is a subject worthy of careful investigation, and if this is carried out the defects of the several most popular methods will be readily discovered, at the cost of the investigator occasionally. Each method possesses peculiar advantages, and each case that is presented for treatment presents peculiar needs. So it must be conceded that any one plan of treatment will not have a general application. A Richmond crown has many advantages, and when the porcelain is faced with metal upon its cutting or masticating surface, it is no doubt the strongest porcelain crown work made, but its disadvantages are not a few. There are many conditions (physiological as well as pathological) which prevent its successful application to some roots. In case of fracture an entirely new piece of work is necessary, and the force necessary to split and remove the cap and pivot is often prejudicial to the future welfare of the root, and the expense of this work is beyond the resources of the ordinary pocket-book.

Where it has been found best to mount Bonwill crowns, I have, in the past year, adopted a method which came so natural that I cannot believe it original, and yet I have seen no hint of another's use of it. I proceed as if it were to be a How four-pin crown, up to the point of cutting off the extra length of screw post. This is cut shorter or left longer, according to the articulation after the crown is ready to set. The alignment of the crown is accomplished before grinding it; then a piece of Field & Mer's thin articulating paper, a little larger than the face of the root, is perforated in the center and slipped over the screw. This serves as a guide in correctly jointing the crown with the root. The crown is then set with amalgam. An improvement upon this method is to band the root very perfectly with coin gold, half the width of the band projecting beyond the face of the root. The cervical portion of the porcelain crown is then fitted inside the band and properly articulated. The rubber-dam is then easily adjusted, the crown is removed, and the face of the root dried. The latter is flooded with carbolic acid for a moment, and thoroughly dried. The crown is set in a well mixed, thick oxy-chloride cement. When this is hard the excess is trimmed off, an angular pit is drilled into the end of the screw in which gold is carefully packed and extended upon the crown. Now in case of fracture of the porcelain, which will sometimes occur, neither post nor band has to be disturbed in the process of setting a new crown. As matters of convenience, comfort and expense, this is of great advantage to both patient and operator.

The swaged gold thimble-caps, made by Dr. E. O. Wilbur, for bicuspids and molars, are the finest of their kind, and in cases where a large mass of gold will not be a disfigurement to the patient, nothing more serviceable can be adjusted to articulate upon. After the root has been prepared internally, the external flange is carefully removed and the outside of the root slightly beveled, and an impression and bite is taken with modeling compound. The model is made of oxy-chloride of zinc, and for the purpose is as good as one of Babbitt, or other metal. Plaster is built upon and around this to represent surrounding teeth taken in the impression. This saves time and annoyance to the patient. A crown is selected, which in size at the neck is smaller than the circumference of the root. The model is slightly trimmed, then with the aid of a light planishing hammer the edge of the crown is drawn out on the arm of the anvil, and with file and engine bur the shaping to the root and the cutting away for the festoons is carefully and accurately done. Trial will prove a tight fit. A How screw-post, of suitable size is now inserted, with repeated trials of the crown over it, and slight nippings from the end of the screw until the crown in its proper position rests on the end of the screw as well as upon the beveled edge of the root. A fine vent hole is then circled over the screw in the crown. The crown is filled with oxy-phosphate and is firmly pressed to position. At a subsequent sitting the cement over the screw is removed, a pit is drilled into the screw and heavy gold is packed into it. These caps being swaged up from a solid piece, the plate is drawn the most over the more prominent points of the die—the cusps—and it is a good plan to drop a little silver solder inside the cap over these thinnest portions before setting them. It would be well if a still larger screw than the largest How screw post should be furnished the profession for the special purpose of setting these caps. They would give still more solidity to the crowns and furnish a larger surface on the ends in which to anchor and weld gold.

A recent case in practice has some points of interest. It is that of a superior central incisor root from which the crown was accidentally broken. Three years ago a wood pivot crown was placed upon the root by another dentist. Six months afterward I found it necessary to substitute a Bonwill crown, using a triangular platinum wire pivot. The root, which was found healthy, had never been sealed at the apex; it was properly treated and sealed before setting the crown. Amalgam as a cement was used. The patient is a vigorous young man of twenty-five, who uses his teeth as he ought to, at least so far as mastication is concerned. The articulation of his teeth is perfect, being very close in front, so close, in fact, that in articulating the crown I found it necessary to grind the palatal surface considerably from the crown after I had burred from the corresponding portion of the root all that was proper. At all events the crown proved weak, and it split off from the pivot about six weeks since. In casting about for the *very best* I could do for my patient I decided upon a Richmond crown. My plan differed from the usual method. It being an unusual case, it called for extraordinary treatment. Through the remains of platinum pivot and amalgam, which was found in excellent condition, the root was drilled and tapped as if a How screw post was to be inserted. After the root was banded and capped with gold, a gold screw two-thirds the diameter of opening in the root was selected; this was wrapped carefully with bibulous paper to the thickness that allowed its easy passage into the root. The end that projected was inked, the cap applied, marked, removed, drilled and readjusted to the root; the screw just passing through the cap was waxed to place, the porcelain crown was ground to band and cap, waxed and the whole carefully removed, invested in plaster and asbestos and soldered.

Herein lies the difference of this from the usual method: the band projects both above and below the cap. The portion in front is cut away in such manner that the crown passes a little inside of it, making a better joint; the soldering is simply of the backing to pins, to the center of the cap, and to the band at *each side.* A few hours after the crown had been set (the threads of the tapped root and screw being well covered) with oxy-phosphate, which had fully hardened, the cap and root back of the crown were drilled, the opening in the root slightly dove-tailed and carefully filled with amalgam, with which the palatal contour was restored, and upon which there was a perfect articulation, the projecting band forming an excellent cup-shaped receptacle for the amalgam.

I cannot conceive of a piece of crown work better calculated to withstand the force of vigorous mastication, and still remain firm.

